# The impact of access to water supply and sanitation on the prevalence of active trachoma in Ethiopia: A systematic review and meta-analysis

**DOI:** 10.1371/journal.pntd.0009644

**Published:** 2021-09-09

**Authors:** Thomas Ayalew Abebe, Gudina Terefe Tucho

**Affiliations:** Department of Environmental Health Sciences and Technology, Jimma University, Jimma City, Oromia, Ethiopia; RTI International, UNITED STATES

## Abstract

**Background:**

Trachoma is a worldwide infectious disease causing blindness. Trachoma continued as a public health problem in Ethiopia due to a lack of sanitation and inadequate prevention strategies. This study aimed to identify the impact of water supply and sanitation intervention on preventing active trachoma among children.

**Methods:**

Systematic literature searches were performed from 4 international databases. The search involved articles published from January 1995 up to March 2019. The Cochran Q and I^2^ statistical tests were used to check heterogeneity among the studies. A random-effect meta-analysis was employed to determine the pooled estimates with a 95% confidence interval (CI). Data analysis was performed using the CMA V.3 and RevMan 5 software program, and the result of the systematic review was reported using the Preferred Reporting Items for Systematic Reviews and Meta-Analyses (PRISMA) guidelines.

**Findings:**

Out of 211 studies screened for the analysis, only 29 studies were finally included in this systematic review and meta-analysis. The result revealed factors that are significantly associated with increased odds of active trachoma. Accordingly, households with no access to toilet facilities (odds ratio [OR]: 2.04, 95% CI: 1.75–2.38), no access to improved water (OR: 1.58, 95% CI: 1.27–1.96), and do not practice regular face washing for children (OR: 4.19, 95% CI: 3.02–5.81) have shown increased odds of active trachoma. Besides, the results show a higher prevalence of active trachoma among children who did not wash their faces with soap and frequently.

**Conclusions:**

The study found strong evidence that lack of access to water, sanitation, and hygiene (WASH) was associated with increased prevalence of active trachoma among children. Therefore, a comprehensive and partnership-oriented program is needed to tackle the problem, but further study will be required to strengthen its implementation.

## 1. Background

Trachoma is the number one cause of infectious blindness worldwide, responsible for an estimated 1.9 million individuals with a visual impairment or blindness [1]. Africa is the hardest-hit continent, with 18 million cases of active trachoma. Fifty percent of the high-risk population for active trachoma was found in 3 countries (Ethiopia, Nigeria, and Malawi) [2,3]. In Ethiopia, 77 million people live in areas where “trachomatous inflammation—follicular” (TF) prevalence in children aged 1 to 9 years was ≥5% [4].

A national Trachoma Action Plan for Ethiopia was conducted in 2012, and elimination of trachoma as a public health problem was planned through Surgery for Trichiasis, Antibiotics, Face washing, and Environmental improvement (SAFE) strategy [5]. Ethiopia has just implemented the second master plan for 2016 to 2020 [6]. However, according to a recent study, the prevalence of active trachoma among children in Ethiopia was ranged between 10.3% [7] and 74.3% [8], which was higher than the World Health Organization (WHO) elimination target [9]. Specific WHO elimination target criteria for active trachoma as a public health problem is a prevalence of TF of <5% among children aged 1 to 9 years in each formerly endemic area. The Antibiotics, Face washing, and Environmental improvement (A, F, and E) components of SAFE are recommended for areas where the prevalence of the sign of active trachoma, TF, is ≥5% in children aged 1 to 9 years [4].

Active trachoma spread through the eye discharge of infected individuals to noninfected people’s eyes through the fingertips, eye-seeking flies, or clothes. Trachoma prevails in places where personal and community hygiene is low, mainly affecting impoverished and disadvantaged community groups. Although the scenarios vary by economic, individual, and environmental reasons, a higher prevalence of active trachoma is associated with several factors. In particular, changes in environmental conditions, most notably access to water, hygiene, and sanitation, are required for its sustainable reductions [10,11].

Most developing countries have limited services to improved water due to longer travel times of more than 30 minutes for round trips and queuing time [12]. The improved drinking water source is a source that is adequately constructed to protect the water from outside contamination, particularly fecal matter. Common examples of improved water sources are piped household water connection, public standpipe, borehole, protected dug wells, protected springs, and rainwater collection. Globally, 207 million people have no access to improved water sources, and sub-Saharan Africa shared two-thirds of its burden. Accordingly, 29.4 million Ethiopians have no access to improved water sources [13].

Access to latrines is defined as those built and owned by households to dispose of human excreta safely. In contrast, latrine use is defined as households having shared or privately owned functioning latrines without visible feces in the vicinity, visible fresh feces inside the latrine pit, and a visible footpath to the latrine [14]. Even though 68% of the Ethiopian population had access to the unimproved latrine [15], half were not using it [14], with 15.9% slippage rate of open defecation free villages [16].

As observed, access to water, sanitation, and hygiene (WASH) service is limited in Ethiopia, while the prevalence of trachoma was still high. Although there are some achievements, there is a need for increased intersectoral collaboration between trachoma and WASH sectors for effective program implementation and efficient resource utilization [17]. However, sufficient data related to the impact of access to WASH on trachoma were not available to support policy and program recommendations. Existing studies on the association between water supply and sanitation are inconsistent and unreliable for policy recommendations. For instance, the study report from the Amhara region shows varying results with a negative (adjusted odds ratio [AOR]: 0.006, 95% confidence interval [CI]: 0.001 to 0.03) [18] and positive (AOR: 1.03, 95% CI: 1.00 to 1.06) association of latrine use with the active trachoma [19].

The association of access to water supply and active trachoma studies also reported varying results. A survey conducted in the Oromia region found that people who walked lower than 15 minutes to fetch water had the highest prevalence of trachoma (AOR: 1.75, 95% CI: 1.26 to 3.03). In contrast, study results from the Amhara region show that individuals living within 30 minutes of walking distance to water sources had lower chances of contracting active trachoma than those traveling over 30 minutes to get water (AOR: 0.02, 95% CI: 0.01 to 0.05) [18]. Furthermore, the study report on the association between facial washing practice and active trachoma in the Amhara region indicates that children who did not wash their faces had 30 times increased odds of active trachoma than their counterparts [20]. Another study from the same region also showed increased odds of active trachoma in children who did not wash their face by 1.72 times [21].

The variations of occurrences of active trachoma and access to water sources between regions or within the region would be due to factors related to the level of socioeconomic development, the level of awareness, sociocultural factors, level of education, and other conditions. Determining the pooled impact of access to water supply, sanitation, and face hygiene at a country level will provide an overall picture with a better estimation to help policy and program reform. Therefore, the objective of this study is to provide comprehensive results on the association of access to water supply, sanitation, and face hygiene with the prevalence of trachoma. The findings from this study can be used for the efficient integration of WASH and trachoma control programs. The result also shows the need for conducting interventional studies and randomized controlled trials that can help develop evidence-based planning.

## 2. Methods

### 2.1 Searching strategy

This study conducted a systematic review and meta-analysis on relevant literature on the impact of WASH intervention on active trachoma published since June 1995. The articles were systematically searched from PubMed, Google Scholar, Science Direct, Embase, and Cochrane Library databases. The study also employed the following search terms: [excre*], [defecat*], [Stool], [faec*], [fec*], [latrine*], [sanitation], [environment*], [toilet*], [water*], [fac*], [wash*], [soap*], [clean*] [sanitation*] [sanit*], [hygiene*] were used separately and combining with Boolean operators such as “OR” or “AND.” Any additional articles found to be pertinent through this review were also considered. The study included articles in the systematic review only if they measured WASH-related indicators, active trachoma infection, and attempted to quantify the association between WASH indicators and active trachoma. The meta-analysis was conducted on specific intervention outcome relationships based on available data. The Preferred Reporting Items for Systematic Reviews and Meta-Analyses (PRISMA) Statement was used for reporting the finding from a systematic review [22]. This systematic review was registered on PROSPERO available online at https://www.crd.york.ac.uk/prospero/display_record.php?RecordID=205199.

### 2.2 Selection criteria and data extraction

The selection of articles for inclusion was made with a dual-step review procedure. First, all identified studies were examined by their titles and abstracts, and those failing the criteria were excluded. After this step, the investigators autonomously reviewed the full text of pertinent articles with a standard protocol. Disagreement concerning the eligibility of a study and inclusion parameters was discussed and resolved based on a consensus. After agreeing on eligible studies, one of the investigators extracted relevant data using a standard protocol. Another investigator ensured extraction reliability and checked the presence of discrepancies. The data extraction sheet was prepared to accommodate the study characteristics such as the author’s name, region, study period, publication year, study design, study setting, sampling method, sample size, active trachoma, or odds ratio (OR). All articles related to WASH and trachoma not conducted in Ethiopia were excluded.

### 2.3 Quality assessment

The study employed the Newcastle–Ottawa quality assessment tool scale amended for cross-sectional studies to check its quality [23]. The quality assessment instrument has 3 measurements. The first part is categorized out of 5 stars and assesses the quality of the methodology of a study. The second measure of the tool ranked out of 3 stars and evaluated the comparability of studies. The final part of the tool was categorized from 2 stars and measured the quality of the original articles and their statistical analyses. Two independent authors evaluated the quality of the original articles using the tool as a protocol. Subsequently, studies with medium quality (satisfy 50% of quality assurance criteria) and high quality (more than 6 out of 10 scales) were considered for the review. One investigator (TAA) performed the quality assessment independently and documented the results in separate tables. The analysis covered all related research, irrespective of their overall quality ranking. Quality scores did not influence the meta-analysis or subsequent description of impact factors. Still, they did help explain the overall consistency of individual studies and define study gaps (S4 Table).

### 2.4 Meta-analysis

Three meta-analyses were conducted using WASH-related indicators such as latrine ownership, access to water, and face washing. Studies with comparable increased ORs of active trachoma were reported. All ORs used in meta-analysis reflected the results of cross-sectional risk factor analyses. The data have been stratified by the most commonly reported indicators (measures) of active trachoma, TF, or trachomatous inflammation—intense (TI). Reported ORs worked as effect measures. We have also incorporated adjusted ORs in the meta-analysis [24]. When ORs were not reported, we calculated from 2 × 2 contingency tables. The study used a Comprehensive Meta-Analysis Version 3 statistical software and Review Manager Version 5.3 to conduct meta-analysis and develop forest plots and meta-regression [25]. The study also investigated publication bias by using funnel plots [26] and Begg and Mazumdar rank correlation test because of the inclusion of many studies in the analysis, regardless of their treatment effect. In contrast, small studies are more likely to be included when they show a relatively significant treatment effect [27]. Heterogeneity between studies was determined using Higgins I^2^ and Cochran Q tests [24]. When heterogeneity was moderate to high (I^2^ > 50%), subgroup analyses were performed to identify potential sources of heterogeneity. Random effects models were used throughout the study to enhance the generalizability of the results [28], and pooled ORs for the effect of the selected WASH-related indicators on active trachoma were employed [24]. Associations between the WASH components and trachoma were aligned so that all ORs reflect the relationship between WASH indicators on active trachoma [29].

## 3. Result

### 3.1 Overview of included studies

A total of 211 studies were identified from the databases, and 150 duplicate articles were excluded after reviewing the titles and abstracts. Additional 32 studies were removed after reading the titles and abstracts based on preset inclusion criteria. Finally, 29 full-text articles were considered based on inclusion criteria predefined for this systematic review (see S1 Fig).

The characteristics of the 29 studies included in this review have been described in S1 Table. All included study designs were cross-sectional, were conducted in different regions of Ethiopia, and with sample size of children aged 1 to 9 years. Accordingly, the sample size for the study ranges from 97 children included from the Southern Nations, Nationalities, and Peoples’ Region (SNNP) to 69,236 from the Amhara region. These studies were conducted from 2007 to 2019. In this meta-analysis, a total of 185,711 people were included to estimate the overall impact of WASH intervention on active trachoma (see S1 Table).

### 3.2 Association between active trachoma and WASH exposure

#### 3.2.1 Association between active trachoma and latrine ownership

From the first meta-analysis in S2 Fig, the forest plot result showed that children from households without latrine had 2.04 times increased odds of active trachoma than those who did have latrine (OR: 2.04, 95% CI: 1.75 to 2.38) and significantly associated with trachoma (*p* < 0.00001). A random-effect meta-analysis was used to estimate the pooled ORs due to considerable heterogeneity among the studies (I^2^ = 92%).

#### 3.2.2 Association between active trachoma and water access

In the second meta-analysis, the result depicted that the increased odds of active trachoma were 1.6 times higher among children from households without access to improved water sources (OR: 1.58, 95% CI: 1.27 to 1.96). A random-effect meta-analysis was also used to estimate the pooled ORs due to considerable heterogeneity among the studies (I^2^ = 92%) (see S3 Fig).

#### 3.2.3 Association between active trachoma and face washing

The third meta-analysis result showed that the increased odds of active trachoma was 4.2 times higher among children who had no regular face washing practice. Face washing practice, and the prevalence of active trachoma, was also significantly associated (*p* < 0.00001). A random-effect meta-analysis was similarly used due to significant heterogeneity I^2^ = 74% as presented in the studies (S4 Fig).

The assessment of publication bias from these 3 meta-analyses, with Begg and Mazumdar rank correlation tests and funnel plot, has shown no statistically significant publication bias or *p* > 0.05 (see S5–S7 Figs).

### 3.3 Subgroup analysis on covariates: A latrine, face washing with soap, face cleanliness of any discharge, washing frequency, and impact of the latrine presence among regions

Identification of sources of heterogeneity was made by 3 subgroup analyses on covariates including a latrine, face washing with soap, face cleanliness of any discharge, washing frequency, and the covariate impact of latrine among regions. The covariate latrine was split into 3 subgroups; latrine access, latrine use, and type of latrine. The next covariate included 5 subgroups: face washing using soap, clean face of any discharge, face washing once per day, face washing twice per day, and face washing thrice per day. The impacts of latrine use among regions were split into 6 subgroups: Oromia, Amhara, Tigray, SNNP, Somali, and Diredawa. Thus, the finding provided us estimates of the impact of latrine on the prevalence of active trachoma among children.

#### 3.3.1 Subgroup analysis on the impact of latrine access, latrine utilization, and type of latrine on active trachoma

Results from the subgroup analysis in S8 Fig or S2 Table show that increased odd of active trachoma was found higher among children from households who did not utilize latrines (OR: 2.64, 95% CI: 1.73 to 4.04) followed by children from households who had no latrines (OR: 2.28, 95% CI: 1.75 to 2.98). We also found statistically significant heterogeneity between subgroups (*p* < 0.00001), which means that the covariate considered (latrine) in the subgroup analysis statistically significantly modifies the impact on the prevalence of active trachoma.

#### 3.3.2 Subgroup analysis based on face washing using soap, clean face of any discharge, face washing once per day, face washing twice per day, and face washing thrice per day

The findings from the subgroup analysis in S9 Fig or S3 Table indicate that children who did not wash their faces found 5.96 times increased odds of active trachoma (OR: 5.96, 95% CI: 2.87 to 12.38) than children who washed their faces more than 3 times a day. Children who did not wash their faces found 5.85 times increased odds of active trachoma (OR: 5.85, 95% CI: 2.84 to 12.04) than those who washed their faces twice a day.

Children with dirty or unclean faces have 4.68 times increased odds of active trachoma (OR: 4.68, 95% CI: 2.44 to 8.99) than children with apparently clean faces. Children who did not use soap during face washing found 3.63 times increased odds of active trachoma (OR: 3.63, 95% CI: 2.51 to 5.25) than those who used soap during face washing. Statistically significant heterogeneity was also found between subgroups (*p* < 0.00001).

#### 3.3.3 Subgroup analysis based on region (impact latrine on active trachoma)

The findings from the subgroup analysis in S10 Fig show that children from households that had no latrines and living in the Diredewa region were associated with increased prevalence of active trachoma (OR: 4.66, 95% CI: 2.77 to 7.84), followed by Amhara, Somali, SNNP, Oromia, and Tigray regions. However, the heterogeneity within subgroups in Diredewa, Tigray, and Somali was not statistically significant.

## 4. Discussion

This review identified the relationship between occurrences of active trachoma with access to water, use of latrine, and washing of a child’s face. The findings revealed that children from households who had no latrines had 2.04 times increased odds of active trachoma than those who had latrine facilities. Therefore, we finally confirmed that latrine ownership is significantly associated with the prevention of active trachoma. Other similar studies revealed an association between latrine presence and reduced odds of active trachoma [30]. Some randomized clinical trials (RCTs) also confirmed the significant association between latrine use and decreased odds of active trachoma [10,31,32]. According to secondary cohort analysis from an RCT in Ethiopia, when latrine use rises by 10%, the prevalence of active trachoma decreases by 2% [31]. Thus, living in communities with high sanitation coverage protects children against active trachoma [29,33,34].

The association between the absence of latrine and increased odds of active trachoma can be explained by the lack of latrine, leading to open defecation, favorably facilitating the breeding of eye-seeking flies (*Musca sorbens*). Openly defecated human feces are the best and comfortable medium for *M*. *sorbens* breeding. So *M*. *sorbens* is the carrier of the bacterium chlamydia trachomatis to the human faces, which is the agent for active trachoma [35,36]. Studies have shown that fly control interventions decrease trachoma transmission by 4-fold [32].

Subgroup analysis indicated that latrine utilization had a higher association with reduced prevalence of active trachoma than latrine access. So latrine utilization was better in protecting active trachoma than latrine accessibility in reducing active trachoma even though both have a protective effect. Furthermore, the finding showed that children from households without latrine access developed higher odds of active trachoma in the Amhara region. Plausible explanations might be related to the high endemicity of the diseases in the region [37] and associated with many studies conducted in the area to alleviate the problems [38,39].

The increased odds of active trachoma among children from households walking more than 30 minutes to collect improve water were 1.6 times higher than those of households having access to improved water at less than 30 minutes within walking distance. Many studies have shown that increased distance to the improved water source was significantly associated with the raised prevalence of active trachoma. The reason was that children were unlikely to wash their faces regularly if the improved water source is far [33,40–44]. Nevertheless, other researchers did not accept this reason, and they reported no association between far distance to get improved water and increased odd of active trachoma. The underlined reason was that the amount of improved water supply to the household can be more concerning than the walking distance to the improved water source [29,45,46]. Other studies rejected both propositions; neither the amount of improved water brought into the house nor the distance traveled to collect improved water impacted reducing active trachoma. However, the amount of improved water used (water allocated daily) for washing children’s faces was significantly associated with lowering active trachoma [47]. Probably, the reason for this is more closely linked to the amount of water used only gives a vague indication of their hygiene or whether or not the water is used can decide on the prevention of active trachoma [48–50].

We found that children who did not wash their faces were 4.2 times increased odds of active trachoma. The increased odds of active trachoma became higher and higher when the frequency of washing face became lower and lower and the children stayed with an uncleaned face of any discharge. Other researchers also provided the most substantial evidence of the relationship between the hygiene factors and trachoma. Their research revealed that the effects of a clean face of any discharge, increased facial washing frequency, and use of soap have all been correlated with lower odds of active trachoma. Our findings have shown that face washing and clean face of any discharge were associated with the reduced odds of active trachoma in children aged 1 to 9 years [29,42,47,51–54].

### 4.1 Policy implications

Trachoma is among the neglected tropical diseases (NTDs) targeted for eliminating the Sustainable Development Goal (SDG) 3 of the SDG by 2030 [55]. It is one of the 20 NTDs, the world’s leading infectious disease causing blindness. It is responsible for the blindness or visual impairment of about 1.9 million of the world’s poorest people [1,56]. Trachoma causes a continued cycle of poverty and inadequate health due to vision loss, which hinders people from working and caring for their families. It primarily affects the most marginalized communities in the developing world without essential services [57]. Ethiopia is one of the hardest-hit countries where trachoma is endemic [3]. According to recent research in Ethiopia, the average prevalence of active trachoma among children was 26.9% [39]. This prevalence was significantly higher than WHO target for eliminating trachoma [2]. Trachoma causes approximately 1.4% of all blindness worldwide and 12% blindness in Ethiopia [37]. Trachomatous blindness is irreversible [2]. Persons with impaired vision tend to experience restrictions on autonomy and mobility and educational achievements. Persons with impaired vision also have a higher risk of falling and injury, poor mental well-being, cognitive impairment, and social isolation [58]. Moreover, visual impairment can affect the typical learning sequence in social, motor, language, and cognitive development and leads to low motivation for exploring the environment, initiating social interaction, and manipulating objects [59].

In trachoma endemic countries like Ethiopia, treatment (Surgery and Antibiotics) has not been sufficient to eliminate trachoma. Still, additional programs should also be planned toward sustainable WASH programs. The current strategy between NTD control programs and those in the WASH sector lead to more substantial efforts at collaboration, coordination, and cooperation between the programs [17,54,60,61].

Ethiopia put many efforts into access to improved sanitation; however, different reports revealed that these gains systematically overlooked the poorest and most marginalized populations, specifically the most susceptible to trachoma [62,63]. Ethiopia is also far from achieving the SDG target of universal access to improved WASH facilities. According to WHO/United Nations International Children’s Emergency Fund (UNICEF) Joint Monitoring Program (JMP) report, Ethiopia was ranked 112th from 117 countries for improved water supply (11%), ranked 95th from 96 countries for improved sanitation facility (6%), and ranked 71st from 78 countries for handwashing facility with soap (8%) [13]. Recent studies show that more than 35.6% of the Ethiopian households practiced open defecation, and, among those who had latrine (unimproved), only 50% of them used it [14,16,64]. In rural Ethiopia, only 57% of households have access to an improved drinking water source. Only 3% of them get piped water into their dwelling or in their neighbor’s yard, and only 7% of households practiced handwashing using soap. Amhara region is among the region having the lowest (5%) handwashing practice with soap. Only 7% of Ethiopian households were practicing appropriate water treatment methods [15]. Moreover, only 37.4% of children in Ethiopia across the regions have practiced face washing twice a day [65].

Government and nongovernmental organizations (NGOs) promote WASH programs to reduce diarrheal diseases, and, as such, few promote face washing alongside handwashing with soap. Our findings support the importance of access to WASH promotion to reduce trachoma. Growing attention to investment in safely managed WASH practices that include daily face washing will support the achievement of the trachoma control program and elimination targets. Household latrine access and use, access to improved water, and face washing practice were important indicators in trachoma prevention and control programs. The continued use of these indicators will help improve health, education, gender equality, and economic productivity by the end of 2030 [3,6,29,61,66]. The achievement of these activities requires an integrated enabling policy involving the participation of relevant sectors.

## 5. Limitations of the study

In this literature review, we found some limitations. The first was how terms like “face washing,” “latrine use,” “latrine access,” and others were defined within papers. The other limitation was the quality of the papers and the cross-sectional nature of the included articles. In addition, the WASH intervention programs varied, leading to high heterogeneity across studies. Nevertheless, these limitations did not pose significant implications for the results of the studies.

## 6. Conclusions

Our study evidenced that the Environmental improvement (E) and Face washing (F) elements of the SAFE strategy or WASH promotions were substantial in controlling active trachoma. Variables such as the absence of a latrine, not using a latrine, lack of improved water access, not washing face frequently, and not using soap during face washing were significantly associated with increased odds of active trachoma. The National Trachoma Prevention and Control and National WASH partnership programs should be strengthened for effective and efficient program implementations. Further randomized controlled trial studies related to WASH intervention are needed to provide evidence supporting effective and enabling integrated programs contributing to reducing the active trachoma.

**Key learning points**

- Active trachoma is the number one cause of infectious blindness.
- Environmental improvement (E) and Face washing (F) elements of the Surgery for Trichiasis, Antibiotics, Face washing, and Environmental improvement (SAFE) strategy were substantial in controlling active trachoma.
- Strong evidence found that lack of access to water, sanitation, and hygiene (WASH) was associated with increased prevalence of active trachoma among children.
- An inclusive and collaborative-oriented program is necessary to eliminate active trachoma.
- Integrated programs (WASH and trachoma control and prevention programs) should be strengthened to reduce active trachoma.

**Top five papers**

- WHO. Report of the 20th meeting of the WHO alliance for the global elimination of trachoma by 2020. The WHO Alliance for The Global Elimination Of Trachoma by 2020; 26–28 April; Sydney, Australia: WHO; 2019. p. 69.
- WHO. Weekly epidemiological record: WHO Alliance for the Global Elimination of Trachoma by 2020: progress report on elimination of trachoma, 2018. 2019.
- Emerson PM, Cairncross S, Bailey RL, Mabey DCW. Review of the evidence base for the ‘F’ and ‘E’ components of the SAFE strategy for trachoma control. Trop Med Int Health. 2000;5(8):515–27.
- Stocks ME, Ogden S, Haddad D, Addiss DG, McGuire C, Freeman MC. Effect of Water, Sanitation, and Hygiene on the Prevention of Trachoma: A Systematic Review and Meta-Analysis. PLoS Med. 2014;11(2):1–30.
- Solomon AW, World Health Organization, London School of Hygiene and Tropical Medicine, International Trachoma Initiative. Trachoma control: a guide for programme managers. 2006.

## Supporting information

S1 Table Summary of publications reporting on WASH-related risk factors for active trachoma in Ethiopia, 2021. WASH, water, sanitation, and hygiene.
(DOCX)

S2 Table Summary of subgroup analysis examining the association of latrine exposures with active trachoma in Ethiopia, 2021. (DOCX)

S3 Table Summary of subgroup analysis of face washing with soap, frequency, and face cleanliness on active trachoma in Ethiopia, 2021. (DOCX)

S4 Table Newcastle–Ottawa quality assessment scale adapted for meta-analysis. (DOCX)

S1 Fig PRISMA flow diagram of included studies. PRISMA, Preferred Reporting Items for Systematic Reviews and Meta-Analyses. (TIF)

S2 Fig Impact of no latrine on prevalence of active trachoma. (TIF)

S3 Fig The impact of inaccessible to water supply on active trachoma. (TIF)

S4 Fig The impact of face washing on active trachoma. (TIF)

S5 Fig Funnel plot for latrine ownership versus trachoma. (TIF)

S6 Fig Funnel plot for water access versus trachoma. (TIF)

S7 Fig Funnel plot for face washing versus trachoma. (TIF)

S8 Fig Random-effect subgroup analysis of impact latrine component on trachoma. (TIF)

S9 Fig Random-effect subgroup analysis of impact face washing with soap, face cleanliness, and washing frequency on trachoma. (TIF)

S10 Fig Random-effect subgroup analysis of impact latrine on trachoma among regions. (TIF)
