# Supplementary material for: The impact of access to water supply and sanitation on the prevalence of active trachoma in Ethiopia: A systematic review and meta-analysis
Source: PLoS Negl Trop Dis. 2021 Sep 9;15(9):e0009644. doi: 10.1371/journal.pntd.0009644 (PMC8428667; doi:10.1371/journal.pntd.0009644)
Supplement: S1 Table — WASH, water, sanitation, and hygiene. (DOCX) [file pntd.0009644.s001.docx]

**S1 Table: Summary of publications reporting on Water, Sanitation, and Hygiene related-risk factors for active trachoma in Ethiopia, 2021.**

| **Sr.No.** | **Reference** | **Study Design** | **Region** | **Study**  **Year** | **Study Population children aged 1-9 years (n=185,711)** | **WASH-related report** | **Type of**  **Trachoma**  **(Active Trachoma)** | **Diagnostic**  **Approach** | **Effect measure** | **Assessment Method** |
| --- | --- | --- | --- | --- | --- | --- | --- | --- | --- | --- |
|  | ([Adamu and Fereji, 2018](#_ENREF_3)) | Cross-sectional | Oromia | Dec-2014 | 961 | L, W, F | TF | Clinical exam | OR | Q, O |
|  | ([Adera et al., 2016](#_ENREF_4)) | Cross-sectional | SNNP | May-2014 | 42,215 | L | TF | Clinical exam | OR | Q, O |
|  | ([Admasu et al., 2015](#_ENREF_5)) | Cross-sectional | SNNP | Mar-2015 | 97 | F | TF | Clinical exam | OR | Q, O |
|  | ([Ahmed et al., 2016](#_ENREF_6)) | Cross-sectional | Amhara | Jan-2015 | 396 | L, F | TF | Clinical exam | OR | Q, O |
|  | ([Alemayehu et al., 2015](#_ENREF_7)) | Cross-sectional | Amhara | Apr-2014 | 215 | L, F | TF | Clinical exam | OR | Q, O |
|  | ([Ali, 2014](#_ENREF_8)) | Cross-sectional | Somali | Jun-2013 | 15,049 | L | TF | Clinical exam | OR | Q, O |
|  | ([Altherr et al., 2019](#_ENREF_9)). | Cross-sectional | Amhara | 2011-2016 | 69,236 | L, W | TF | Clinical exam | OR | Q, O |
|  | ([Anteneh and Getu, 2016](#_ENREF_10)) | Cross-sectional | Amhara | Apr-2015 | 601 | L, W, F | TF | Clinical exam | OR | Q, O |
|  | ([Asres et al., 2016](#_ENREF_11)) | Cross-sectional | Amhara | Dec-2014 | 586 | L, F | TF | Clinical exam | OR | Q, O |
|  | ([Ayalew, 2016](#_ENREF_12)) | Cross-sectional | Oromia | Mar-2015 | 644 | L, F | TF | Clinical exam | OR | Q, O |
|  | ([Basha et al., 2020](#_ENREF_15)) | Cross-sectional | Amhara | May-2018 | 312 | L, F | TF | Clinical exam | OR | Q, O |
|  | ([Bero et al., 2016](#_ENREF_19)) | Cross-sectional | Oromia | Jul-2014 | 41,642 | L, W, | TF | Clinical exam | OR | Q, O |
|  | ([Cumberlanda et al., 2005](#_ENREF_25)) | Cross-sectional | 3 region* | May-2002 | 1960 | L, W, F | TF/TI | Clinical exam | OR | Q, O |
|  | ([Ejigu et al., 2013](#_ENREF_29)) | Cross-sectional | Oromia | Feb-2011 | 346 | L, W, F | TF | Clinical exam | OR | Q, O |
|  | ([Ferede et al., 2017](#_ENREF_34)) | Cross-sectional | Amhara | Feb-2015 | 681 | L, W, F | TF | Clinical exam | OR | Q, O |
|  | ([Gedefaw et al., 2013](#_ENREF_41)) | Cross-sectional | Amhara | Mar-2012 | 409 | L, F | TF | Clinical exam | OR | Q, O |
|  | ([Golovaty et al., 2007](#_ENREF_42)) | Cross-sectional | Amhara | Jul-2007 | 782 | L, W, F | TF/TI | Clinical exam | OR | Q, O |
|  | ([Kassaw et al., 2019](#_ENREF_52)) | Cross-sectional | Amhara | Apr-2017 | 596 | L, F | TF | Clinical exam | OR | Q, O |
|  | ([Kassim et al., 2019](#_ENREF_53)) | Cross-sectional | Oromia | May-2017 | 406 | L, W, F | TF | Clinical exam | OR | Q, O |
|  | ([Ketema et al., 2012](#_ENREF_54)) | Cross-sectional | Amhara | Apr-2012 | 792 | L, W, F | TF/TI | Clinical exam | OR | Q, O |
|  | ([Lemma, 2001](#_ENREF_56)) | Cross-sectional | Amhara | Mar-1997 | 560 | L, F | TF/TI | Clinical exam | OR | Q, O |
|  | ([Mesfin et al., 2006](#_ENREF_61)) | Cross-sectional | Tigray | May-2003 | 1526 | L | TF/TI | Clinical exam | OR | Q, O |
|  | ([Mohamed et al., 2019](#_ENREF_64)) | Cross-sectional | DireDawa | Mar-2019 | 823 | L, W | TF | Clinical exam | OR | Q, O |
|  | ([Nigusie et al., 2015](#_ENREF_68)) | Cross-sectional | Amhara | Jun-2014 | 618 | L, W, F | TF | Clinical exam | OR | Q, O |
|  | ([Oswald et al., 2017](#_ENREF_69)) | Cross-sectional | Amhara | 2011-2014 | 62, 869 | L | TF | Clinical exam | OR | Q, O |
|  | ([Reda et al., 2020](#_ENREF_72)) | Cross-sectional | Tigray | Mar-2018 | 502 | L, F | TF | Clinical exam | OR | Q, O |
|  | ([Tadesse et al., 2017](#_ENREF_79)) | Cross-sectional | Amhara | Dec-2014 | 725 | L, W | TF | Clinical exam | OR | Q, O |
|  | ([WoldeKidan et al., 2019](#_ENREF_93)) | Cross-sectional | SNNP | Apr-2018 | 574 | L | TF | Clinical exam | OR | Q, O |
|  | ([Zerihun, 1997](#_ENREF_95)) | Cross-sectional | Oromia | Jan-1995 | 2457 | L, W | TF/TI | Clinical exam | OR | Q, O |
|  | O, observed directly; Q, questionnaire; and * three regions for Oromia, Amhara, and SNNP; W: water, L: Latrine; F: Face washing; OR: Odd Ratio  TF: Trachomatous inflammation-Follicular; TI: Trachomatous inflammation-Intense | | | | | | | | | |
