# Supplementary material for: The impact of access to water supply and sanitation on the prevalence of active trachoma in Ethiopia: A systematic review and meta-analysis
Source: PLoS Negl Trop Dis. 2021 Sep 9;15(9):e0009644. doi: 10.1371/journal.pntd.0009644 (PMC8428667; doi:10.1371/journal.pntd.0009644)
Supplement: S2 Table — (DOCX) [file pntd.0009644.s002.docx]

**S2 Table: Summary of subgroup analysis examining the association of latrine exposures with active trachoma in Ethiopia, 2021.**

| **Sr. No.** | **Latrine exposure** | **Active trachoma** | | |
| --- | --- | --- | --- | --- |
|  |  | n | Random Effects Pooled OR (95% CI) | I^2^ (95% CI) |
| **1.1.1** | No Access to Latrine | 18 | 2.28 (1.75-2.98) | 92% |
| **1.1.2** | Not Utilize Latrine | 9 | 2.64 (1.73-4.04) | 92% |
| **1.1.3** | Unimproved Latrine type | 1 | 1.19 (1.02-1.39) | NA |
