# Supplementary material for: The impact of access to water supply and sanitation on the prevalence of active trachoma in Ethiopia: A systematic review and meta-analysis
Source: PLoS Negl Trop Dis. 2021 Sep 9;15(9):e0009644. doi: 10.1371/journal.pntd.0009644 (PMC8428667; doi:10.1371/journal.pntd.0009644)
Supplement: S3 Table — (DOCX) [file pntd.0009644.s003.docx]

**S3 Table: Summary of subgroup analysis of face washing with soap, frequency and face cleanliness on active trachoma in Ethiopia.**

| **Sr. No.** | **Face related covariates** | **Active trachoma** | | |
| --- | --- | --- | --- | --- |
|  |  | n | Random Effects Pooled OR (95% CI) | I^2^ (95% CI) |
| **1.1.2** | Had no face washing with soap | 3 | 3.63 (2.51-5.25) | 0% |
| **1.1.3** | Unclean (dirty) face | 7 | 4.68 (2.44-8.99) | 84% |
| **1.1.4** | Had no face washing once/day | 3 | 1.86 (1.33-2.61) | 0% |
| **1.1.5** | Had no face washing twice/day | 6 | 5.85 (2.84-12.04) | 69% |
| **1.1.6** | Had no face washing thrice/day | 1 | 5.96 (2.87-12.38) | 74% |
