# Supplementary material for: The impact of access to water supply and sanitation on the prevalence of active trachoma in Ethiopia: A systematic review and meta-analysis
Source: PLoS Negl Trop Dis. 2021 Sep 9;15(9):e0009644. doi: 10.1371/journal.pntd.0009644 (PMC8428667; doi:10.1371/journal.pntd.0009644)
Supplement: S4 Table — (DOCX) [file pntd.0009644.s004.docx]

**S4 Table: Newcastle-Ottawa Quality Assessment Scale adapted for meta-analysis.**

| **Sr.No.** | **Author** | **Title of the paper** | **Representativeness of**  **the Sample** | **Sample size** | **Non-respondents** | Ascertainment of the exposure | The Subjects in Different Outcome Groups are Comparable | Assessment of the Outcome | Statistical Test | Quality |
| --- | --- | --- | --- | --- | --- | --- | --- | --- | --- | --- |
|  | (Adamu and Fereji, 2018) | Prevalence of active trachoma among children between 1-9 years, in Woliso Town, Central Ethiopia | * | * | * | ** | * |  | * | Good |
|  | (Adera et al., 2016) | Prevalence of and Risk Factors for Trachoma in Southern Nations, Nationalities, and Peoples’ Region, Ethiopia: Results of 40 Population-Based Prevalence Surveys Carried Out with the Global Trachoma Mapping Project | * | * | * | ** | * |  | * | Good |
|  | (Admasu et al., 2015) | Prevalence of Trachoma and Associated Risk Factors among Yello Elementary School Students, In Loma Woreda, Dawro Zone, Ethiopia, 2015 | * | * | * | * |  |  | * | Satisfactory |
|  | (Ahmed et al., 2016) | Prevalence of Active Trachoma and its Associated Factors Among Children Aged 1-9 Years in Dessie City Administration, Amhara Region, Ethiopia 2015 | * | * | * | * | * |  | * | Satisfactory |
|  | (Alemayehu et al., 2015) | Prevalence of Active Trachoma and Its Associated Factors among Rural and Urban Children in Dera Woreda,Northwest Ethiopia: A Comparative Cross-Sectional Study | * | * | * | ** | * |  | * | Good |
|  | (Ali, 2014) | Trachoma Risk Factors and Control Strategy in Somali Regional State, Ethiopia | * | * | * | ** | * |  | * | Good |
|  | (Altherr et al., 2019). | Associations between Water, Sanitation and Hygiene (WASH) and trachoma clustering at aggregate spatial scales, Amhara, Ethiopia | * | * |  | ** | * |  | * | Satisfactory |
|  | (Anteneh and Getu, 2016) | Prevalence of active trachoma and associated risk factors among children in Gazegibela district of Wagehemra Zone, Amhara region, Ethiopia: community-based cross-sectional study | * | * | * | ** | * |  | * | Good |
|  | (Asres et al., 2016) | Prevalence and Risk Factors of cƟǀĞ Trachoma among Children in Gondar Zuria District North Gondar, Ethiopia | * | * | * | ** | * |  | * | Good |
|  | (Ayalew, 2016) | Prevalence Of Trachoma And Associated Factors Of Children Aged 1-9 Years In Community Led Total Sanitation And Hygiene Triggered Village And None Triggered In Girar Jarso  Woreda, North Shoa, Oromia, Ethiopia. | * | * | * | ** | * |  | * | Good |
|  | (Basha et al., 2020) | Prevalence and risk factors of active trachoma among primary school children of Amhara Region, Northwest Ethiopia | * | * | * | * | * |  | * | Satisfactory |
|  | (Bero et al., 2016) | Prevalence of and Risk Factors for Trachoma in Oromia Regional State of Ethiopia: Results of 79 Population-Based Prevalence Surveys Conducted with the Global Trachoma Mapping Project | * | * | * | ** | * |  | * | Good |
|  | (Cumberlanda et al., 2005) | Active trachoma in children aged three to nine years in rural communities in Ethiopia:  prevalence, indicators and risk factors | * | * | * | ** | * |  | * | Good |
|  | (Ejigu et al., 2013) | Rapid Trachoma Assessment in Kersa District, Southwest Ethiopia | * | * | * | ** |  |  | * | Satisfactory |
|  | (Ferede et al., 2017) | Prevalence and determinants of active trachoma among preschool-aged children  in Dembia District, Northwest Ethiopia | * | * | * | ** | * |  | * | Good |
|  | (Gedefaw et al., 2013) | Current state of active trachoma among elementary school students in the context of ambitious national growth plan: The case of Ethiopia | * | * | * | ** | * |  | * | Good |
|  | (Golovaty et al., 2007) | Access to Water and Latrine Facilities in Relation to Active Trachoma Infection in Northern Ethiopia | * | * | * | ** | * |  | * | Good |
|  | (Kassaw et al., 2019) | Prevalence and risk factors of active trachoma among rural preschool children in Wadla district, Northern Ethiopia | * | * | * | ** | * |  | * | Good |
|  | (Kassim et al., 2019) | Prevalence of active trachoma and associated risk factors among children of the pastoralist population in Madda Walabu rural district, Southeast Ethiopia: a community-based cross-sectional study | * | * | * | ** | * |  | * | Good |
|  | (Ketema et al., 2012) | Active trachoma and associated risk factors among children in Baso Liben District of East  Gojjam, Ethiopia | * | * | * | ** | * |  | * | Good |
|  | (Lemma, 2001) | Prevalence and Risk Factors of Trachoma among Children of Woreillu Woreda, South Wollo Administrative Zone | * | * | * | ** | * |  | * | Good |
|  | (Mesfin et al., 2006) | A Community-Based Trachoma Survey:Prevalence and Risk Factors in the Tigray Region of Northern Ethiopia | * | * | * | ** | * |  | * | Good |
|  | (Mohamed et al., 2019) | Trachoma and Associated Factors among School Age Children 4-9 Years in Dire Dawa Administration, Eastern Ethiopia | * | * | * | ** | * |  | * | Good |
|  | (Nigusie et al., 2015) | Prevalence and associated factors of active trachoma among childeren aged  1–9 years in rural communities of Gonji Kolella district, West Gojjam zone, North West Ethiopia | * | * | * | ** | * |  | * | Good |
|  | (Oswald et al., 2017) | Active trachoma and community use of sanitation, Ethiopia | * | * | * | ** | * |  | * | Good |
|  | (Reda et al., 2020) | Prevalence and associated factors of active trachoma among 1–9 years old children in  Deguatemben, Tigray, Ethiopia, 2018: community cross-sectional study | * | * | * | ** | * |  | * | Good |
|  | (Tadesse et al., 2017) | The burden of and risk factors for active trachoma in the North and South Wollo  Zones of Amhara Region, Ethiopia: a crosssectional study | * | * | * | ** | * |  | * | Good |
|  | (WoldeKidan et al., 2019) | Prevalence of active trachoma and associated factors among children aged 1 to 9 years in rural communities of Lemo district, southern Ethiopia: community based cross sectional study | * | * | * | ** | * |  | * | Good |
|  | (Zerihun, 1997) | Trachoma in Jimma Zone, SouthWestern Ethiopia. | * | * | * | ** |  |  | * | Satisfactory |
|  | We assigned stars to evaluate study quality, with nine to ten stars indicating “very good” quality, seven to eight stars indicating “good” quality, five to six stars indicating “satisfactory” quality, and zero to four stars indicating “unsatisfactory” quality. | | | | | | | | | |
