# Supplementary figures and images for: The impact of access to water supply and sanitation on the prevalence of active trachoma in Ethiopia: A systematic review and meta-analysis
Source: PLoS Negl Trop Dis. 2021 Sep 9;15(9):e0009644. doi: 10.1371/journal.pntd.0009644 (PMC8428667; doi:10.1371/journal.pntd.0009644)

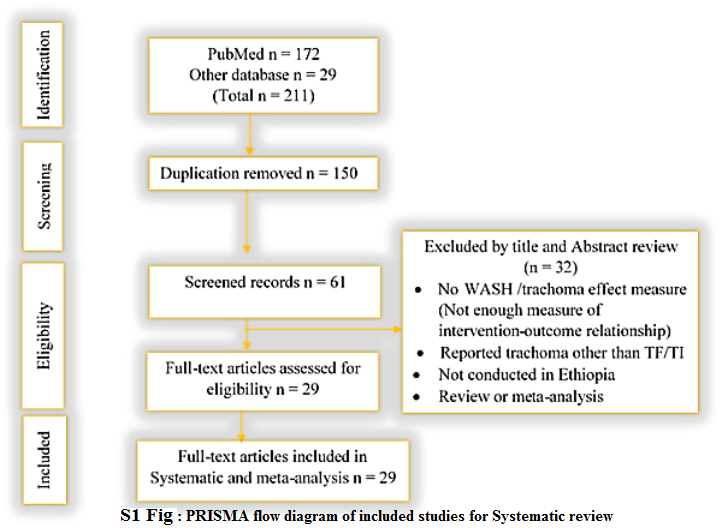

Supplement: S1 Fig — (TIF) [file pntd.0009644.s005.tif]

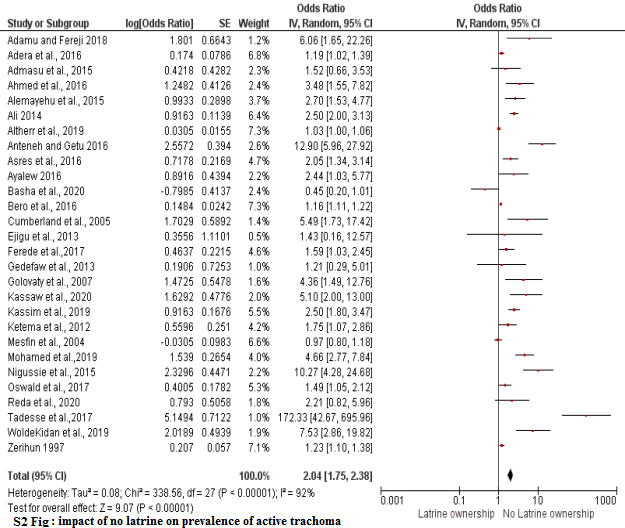

Supplement: S2 Fig — (TIF) [file pntd.0009644.s006.tif]

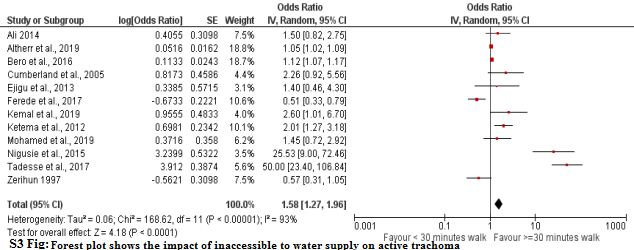

Supplement: S3 Fig — (TIF) [file pntd.0009644.s007.tif]

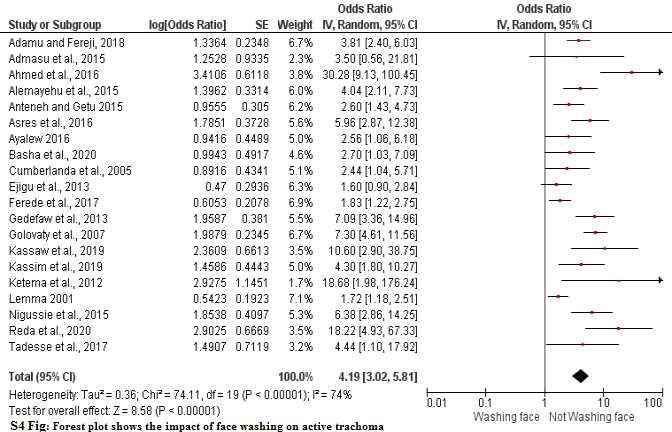

Supplement: S4 Fig — (TIF) [file pntd.0009644.s008.tif]

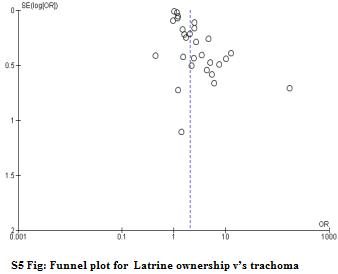

Supplement: S5 Fig — (TIF) [file pntd.0009644.s009.tif]

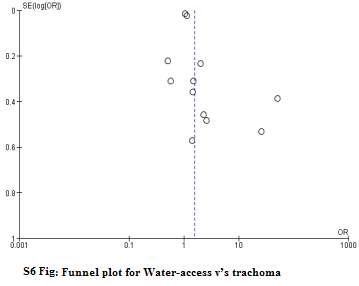

Supplement: S6 Fig — (TIF) [file pntd.0009644.s010.tif]

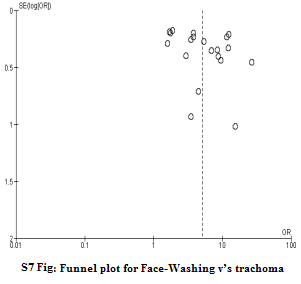

Supplement: S7 Fig — (TIF) [file pntd.0009644.s011.tif]

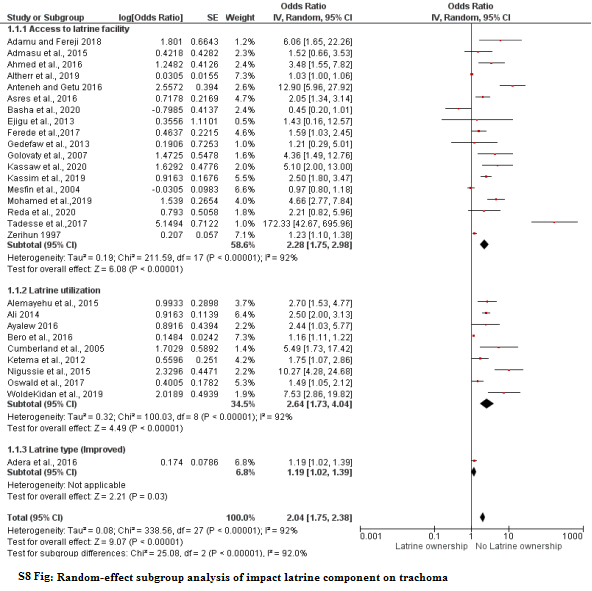

Supplement: S8 Fig — (TIF) [file pntd.0009644.s012.tif]

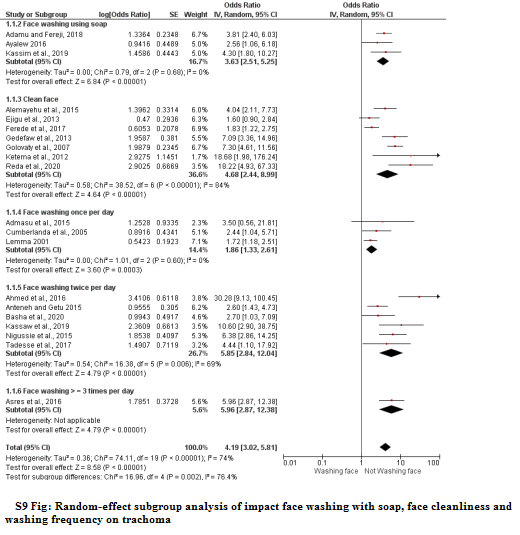

Supplement: S9 Fig — (TIF) [file pntd.0009644.s013.tif]

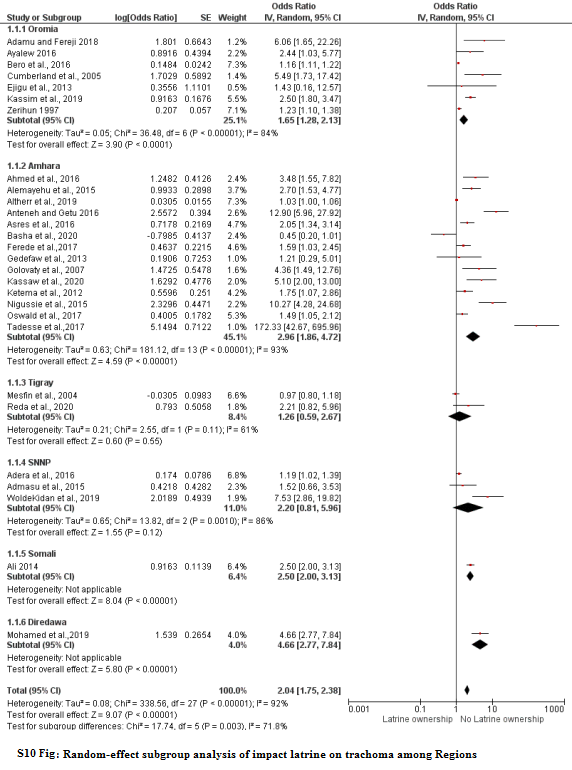

Supplement: S10 Fig — (TIF) [file pntd.0009644.s014.tif]
